# The Effect of Renin-Angiotensin-Aldosterone System Blockade Medications on Contrast-Induced Nephropathy in Patients Undergoing Coronary Angiography: A Meta-Analysis

**DOI:** 10.1371/journal.pone.0129747

**Published:** 2015-06-17

**Authors:** Zhijun Wu, Huan Zhang, Wei Jin, Yan Liu, Lin Lu, Qiujing Chen, Ruiyan Zhang

**Affiliations:** 1 Department of Cardiology, Ruijin Hospital, Shanghai Jiao Tong University School of Medicine, Shanghai, People’s Republic of China; 2 Division of Nephrology, Changhai Hospital, Second Military Medical University, Shanghai, People’s Republic of China; Max-Delbrück Center for Molecular Medicine (MDC), GERMANY

## Abstract

**Background:**

Contrast-induced nephropathy (CIN) is the main complication of contrast media administration (CM) in patients undergoing coronary angiography (CAG) and percutaneous coronary intervention (PCI). There are inconsistent results in the literature regarding the effect of renin-angiotensin-aldosterone system (RAAS) blockers (angiotensin-converting enzyme inhibitors [ACEIs] and angiotensin receptor blockers [ARBs]) on CIN. We evaluated the association between the administration of ACEI/ARBs and CIN, as well as the effect of ACEI/ARBs on post-procedural changes in renal function index, in patients undergoing CAG.

**Methods:**

We searched Pubmed, EMBASE, Cochrane Central Register of Controlled Trials and ClinicalTrials.gov for relevant studies. The primary search generated 893 potentially relevant articles. A total of 879 studies were excluded because they did not meet the selection criteria. Finally, 14 studies were eligible for inclusion. There were 7,288 patients that received ACEI/ARBs and 8,159 patients that received placebo or naive to ACEI/ARBs in the study. A random or a fixed effect model was used to calculate the pooled odd ratios (ORs).

**Results:**

The risk of CIN was significantly increased in the ACEI/ARBs group compared to the control group (OR= 1.50, 95%CI: 1.03-2.18, P =0.03). The magnitude of association was significantly reinforced in the observational studies (OR=1.84, 95%CI 1.19-2.85, P=0.006) but not in the randomized controlled trials (OR=0.88, 95%CI 0.41-1.90 P=0.74). The summary adjusted OR of 4 observational studies was 1.56 (95%CI 1.25-1.94, P<0.0001) and was weaker than the unadjusted OR.

**Conclusions:**

Although there is some evidence to suggest that the administration of RAAS blockers was associated with the increased risk of CIN in patients undergoing CAG, the robustness of our study remains weak. The results are based on small observational studies and need further validation.

## Introduction

The high prevalence of coronary artery disease (CAD) results in a significant increase of coronary angiography (CAG) and percutaneous coronary intervention (PCI). Contrast-induced nephropathy (CIN), also known as contrast- induced acute kidney injury (CI-AKI), is a common and serious adverse reaction of intra-arterial administration of iodinated radiographic contrast medium (CM) [1]. CIN manifests as an asymptomatic acute deterioration in renal function that can be measured within 24 hours of CM administration. Serum creatinine (Scr) levels begin to increase at 24 to 72 hours, peak within 3–5 days, and then return to baseline within 10–14 days. CIN incidence has been calculated to be below 2% in the general population, but above 50% in high-risk patients, such as preexisting chronic renal impairment, diabetes mellitus (DM), congestive heart failure (CHF), and concomitant nephrotoxic exposure [2]. CIN is one of the major causes of hospital-acquired acute renal failure [3] and has been associated with an accelerating rate of chronic kidney disease (CKD) progression and major cardiac adverse events, resulting in dramatically lengthened hospitalization stays, increased morbidity and mortality[4, 5]. A retrospective analysis involving in 7,586 patients receiving PCI (254 experienced CIN) found that CIN was significantly associated with death during the index hospitalization. Among hospital survivors with CIN, 1- and 5- year estimated mortality rates were 12.1% and 44.6%, much greater than those in patients without CIN[6].

With the increasing number of CAG and PCI, more patients are now exposed to CM and are at risk for CIN than before. The unfavorable prognosis of CIN makes the prevention more important because there are no effective treatment options for CIN. Some preventive interventions such as intravenous hydration [7], acetylcysteine [8] and utilization of less nephrotoxic contrast [9] have been used to reduce CIN post-PCI.

The mechanisms of CIN are still poorly understood, involving complex multi-factorial physiopathology with a high inter-individual variability of the nephrotoxic effect of CM. There have been several problems regarding the role of the rennin-angiotensin- aldosterone system (RAAS) blockade medications including angiotensin-converting enzyme inhibitors (ACEIs) and angiotensin receptor blockers (ARBs), in the development of CIN. ACEIs and ARBs are generally administered in patients with cardiovascular diseases (e.g., hypertension, CAD, CHF and ischemic cardiomyopathy) [10–12] and with nephropathy (e.g., diabetic nephropathy, CKD and IgA nephropathy) [13, 14]. Whether ACEIs and ARBs increase or decrease the risk of CIN after CAG and PCI is still unclear. There are contradictory results concerning the use of ACEIs and ARBs before administration of CM imaging. Some studies have indicated that RAAS blockades are effective in reducing of the CIN incidence [15], while others have suggested that the administration of ACEIs and ARBs are an independent risk of CIN [16–19]. This contradiction may be due to significant differences in ethnic background, study design, interventions and relative small sample sizes. Meta-analysis offers an opportunity to estimate and combine the results from multiple studies. The aim of meta-analysis is to increase the statistical power and to improve the estimate of the effect size [20]. Here, we performed a meta-analysis of these trials to systematically estimate the effect of ACEIs and ARBs on the CIN incidence.

## Methods

### Search strategy

The literature search was conducted on PubMed, EMBASE, Cochrane Central Register of Controlled Trials and ClinicalTrials.gov electronic databases to identify the studies; the last search update in December 2014. ACEIs of interest included perindopril, captopril, enalapril, lisinopril, fosinopril and ramipril and ARBs of interest included valsartan, telmisartan, losartan, irbesartan, azilsartan, candesartan and olmesartan. The following keywords and MeSH terms were applied: ‘angiotensin-converting enzyme inhibitor’ or ‘ACEI’ or ‘perindopril’ or ‘captopril’ or ‘enalapril’ or ‘lisinopril’ or ‘fosinopril’ or ‘ramipril’, ‘angiotensin receptor blocker’ or ‘ARB’ or ‘valsartan’ or ‘telmisartan’ or ‘losartan’ or ‘irbesartan’ or ‘azilsartan’ or ‘candesartan’ or ‘olmesartan’, ‘renin-angiotensin-aldosterone’ or ‘RAAS’, ‘contrast-induced nephropathy’ or ‘CIN’, ‘contrast- induced acute kidney injury’ or ‘CI-AKI’, ‘coronary artery disease’, ‘coronary angiography’, ‘percutaneous coronary intervention’ and ‘contrast media’. Additional studies were retrieved from conference proceedings. A manual search and a review of bibliographies from the accessed researches, reviews, and previous meta-analysis were also performed for sources of potential information. If necessary, we contacted the corresponding authors by e-mail to require original data and to avoid duplicating the patients recruited in more than one trial from the same area.

### Selection criteria

The PICO (population, intervention, comparison, and outcome) approach was used to establish the inclusion criteria for the meta-analysis [21]. The population of interest involved patients undergoing CAG. All the patients received CM during the procedure of CAG. The intervention of interest was the administration of ACEI/ARBs or placebo. The comparison was made between the ACEI/ARBs group versus the non-ACEI/ARBs group. The primary outcome was CIN incidence, defined as an absolute increase in Scr values (from 0.3 to 2.0 mg/dL) or by a relative increase as compared with the baseline value (from 25% to 50%) within 2 to 5 days after exposure to CM [2, 22]. The potentially relevant outcomes in renal function before and after exposure to CM were also assessed and included: Scr, estimated glomerular filtration rate (eGFR) and blood urea nitrogen (BUN). The eGFR was calculated based on the Scr using two methods: the Modified of Diet in Renal Disease (MDRD) study formula [23] and the Cockcroft-Gault formula [23, 24]. The following criteria were adopted when estimating studies: (1) clinical trials of human and adults (over 18 years of age) without ethnic or language restriction; (2) ACEI/ARBs administration or placebo; (3) studies incorporating preventive CIN interventions were included only if both arms took preventive measures; (4) studies reporting the CIN incidence in both arms; (5) eligibility according to renal function was not restricted; (6) controlled trials with full text articles or conference abstracts; (7) if more than one article of the same trial existed, only the most recent or the most complete article was included; (8) trials using crossover design or lacking washout period were excluded.

### Data extraction

The PRISMA (Preferred Reporting Items for Systematic Reviews and Meta-Analyses) guideline was followed in the current meta-analysis [25, 26] (S1 Table). Two of the authors (WZ and ZH) independently extracted key information from the individual eligible studies. Differences over inclusion of studies were resolved through discussion in order to reach a consensus. This process was supervised by the third author (ZR). If required, we would consult with all the other authors (JW, LY, LL, CQ and ZR). A decision was made based on consensus. The following characteristics were used to describe each study: first author’s name, publication year, ethnicity, country of origin, study design, number of participants, CM usage, ACEI/ARB and other drugs, endpoints, age, gender, mean and standard deviation (SD) of the renal function index (Scr, eGFR and BUN) before and after exposure to CM.

### Risk of bias assessment

Two authors (WZ and ZH) independently appraised the study-level risk of bias. Disagreements in ratings were resolved by discussion and consensus. We used a modified version of Cochrane Collaboration’s tool[27] to assess the risk of bias of randomized controlled trial (RCT). The revised tools rates 7 domains: selection bias (random sequence generation and allocation concealment), performance bias (blinding of participants and personnel), detection bias (blinding of outcome assessment), attrition bias (incomplete outcome data), reporting bias (selective reporting) and other bias. The risk of bias was reported as ‘low risk’, ‘unclear risk’, or ‘high risk’.

As for the observational studies, the Newcastle-Ottawa Scale (NOS) [28, 29] was used based on three broad perspectives: Selection, Comparability, and Exposure. A study can be awarded a maximum of one star for each numbered item with the Selection and Exposure categories, and a maximum of two stars for Comparability. Studies can be awarded a maximum score of 9 stars with scores of 5 stars or more regarded to be of medium to high study quality.

### Statistical analysis

Continuous variables were expressed as mean ± SD or median (5th and 95th percentiles). Standard error was converted to SD. Dichotomous data were analyzed using odd ratios (ORs) corresponding to the 95% confidence interval (CI). Quantitative outcomes, changing from baseline to post-catheterization, were summarized and assessed by the mean difference (MD) with 95% CI between the ACEI/ARBs and control groups. A fixed-effects model was used to estimate the effect size for homogeneous studies and a random-effects model was used for heterogeneous studies [30]. Statistical heterogeneity on each outcome of interest was assessed using the Chi-square-based Q statistic test [31] and quantified by means of inconsistency index I^2^ statistic. A P value for heterogeneity exam >0.1 indicated no significant statistical heterogeneity. The I^2^ statistic, ranging from 0% to 100%, was defined as the percentage of the observed between-trial variability in effect size due to heterogeneity rather than chance. A high value of I^2^ implied that heterogeneity accounted for most of the between-study variation [32, 33]. The significance level of the combined ORs, which was estimated by the Z test, was of P＜0.05. If the SD of the renal function index at baseline or post-catheterization were not given, the median SD from all the other studies was used.

To further detect clinically significant heterogeneity, predefined subgroup analyses were conducted a priori according to the study type (randomized controlled trial [RCT] and observational study) and the ethnicity subtypes (Asians, Indians and Caucasians). Sensitivity analyses were performed to evaluate the contribution of individual studies to combined effect estimates by sequentially removing each study one at a time. In addition, meta-regression analyses were conducted to estimate the extent to which one or more study-level variables, explained the heterogeneity of combined effect estimates of ACEI/ARBs on the CIN incidence. The visual funnel plot and Egger’s linear regression test were used to estimate publication bias. P< 0.10 was defined as significant for the Egger’s test. An asymmetric plot suggests the possible presence of publication bias, which can be verified by a T-test. P<0.05 was regarded as statistically significant with the exceptions of I^2^ and Egger’s statistics [34]. Data management and statistical analysis were performed using Stata 11.0 (Stata Corporation, College Station, TX, USA). All P values were 2-sided.

## Results

### Included articles and study characteristics

**Fig 1:**
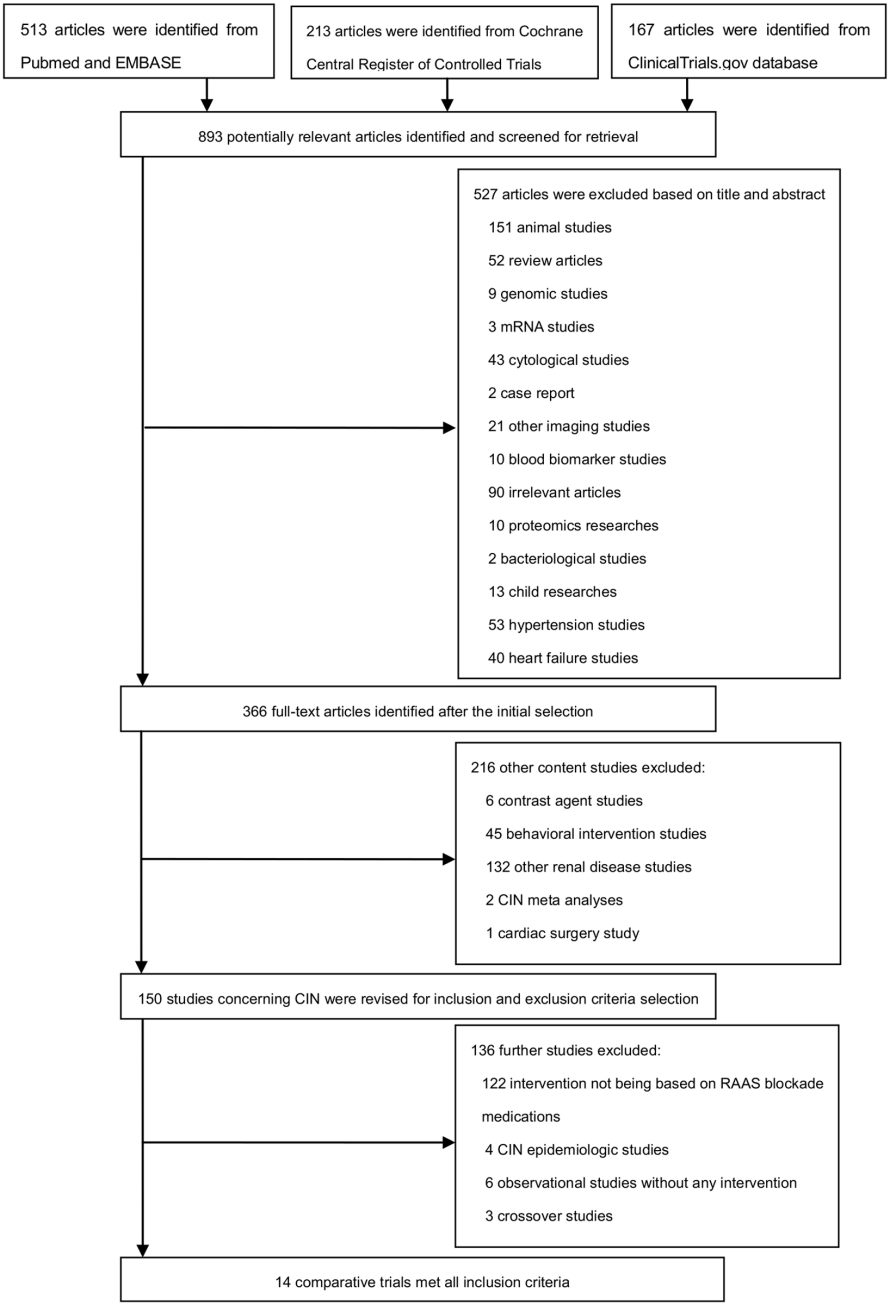
Flowchart of meta-analysis study selection.

A flow diagram schematizing the stepwise selection process of selecting and excluding articles is illustrated in Fig 1. The primary search for clinical trials concerning ACEI/ARBs and CIN generated 893 potentially relevant articles using the above strategies (Pubmed and EMBASE: n = 513, Cochrane Central Register of Controlled Trials: n = 213, Clinical Trials.gov: n = 167). Of these, 527 were excluded because they did not meet the selection criteria. The remaining 366 results were considered to be of relevance and full papers were carefully screened. Fourteen studies published between 1999 and 2013 were deemed adequate for the selection criteria. There was good agreement between the authors on the inclusion of the selected trials. The study design, the feature of the interventions, the baseline renal function and the primary endpoints are summarized in Table 1. The final population for the meta-analysis was composed of 15,447 patients (7,288 treated with ACEIs or ARBs and 8,159 in the control group) [15–19, 35–43]. The baseline characteristics of the 14 trials included in the meta-analysis are summarized in Tables 1 and 2. There were 10 studies performed in Caucasians [16–18, 35, 37–42] and 3 in East Asians [15, 19, 43]. Besides, the study by Gupta et al. [36] that recruited a randomized Indian population was regarded as an independent subgroup because its lineage was complicated and could not be simply grouped as Asian or Caucasian [44–46]. Seven studies received ACEIs, such as captopril and benazpril [15–17, 36, 37, 40, 41], 1 study received valsartan [38] and 6 studies received either ACEIs or ARBs [18, 19, 35, 39, 42, 43]. Seven random controlled trails (RCTs) were identified in the meta-analysis [15, 36–41], while the other 7 studies were observational studies (i.e. cohort, cross-sectional or case-control studies) [16–19, 35, 42, 43]. Ten studies used a prospective study design [15, 16, 18, 35–41] and the other 4 studies were retrospective studies [17, 19, 42, 43]. Most studies regarded CIN as an increase in Scr of ≥0.5 mg/dL or ≥25% or 50% from baseline within 24-72h after CM exposure, except the study by Rim et al. (CIN was defined as an absolute increase in Scr of ≥0.3 mg/dL or ≥50% with 48h post-catherization) [19]. All the patients received low-osmolar or iso-osmolar CM when undergoing CAG or PCI. CM volume ranged from 60 ml to 225 ml. Almost all of the patients received preventive treatment for CIN (i.e. hydration with isotonic saline infusion, intravenous sodium bicarbonate and N-acetylcysteine). Two studies enrolled patients with mild to moderate renal function impairment [15, 16], while the study by Rosenstock et al. involved patients with CKD stages 3–4 [39].

**Table 1:** The baseline charcteristics of all qualified studies included in the meta-analysis.

First Author	Year	Ethnicity	Country	Study design	Drug	Study type	Baseline renal function describtion	Primary Endpoints	Status	Sample size, number	Number of CIN
Barış N	2012	Caucasian	Turkey	prospective	ACEI/ARB	observational	eGFR≥ 30mL/min	CIN, ΔScr>0.5mg/dL or >25% within 48-72h post-catherization	case	200	35
									control	95	7
Cirit M	2006	Caucasian	Turkey	prospective	ACEI	observational	≥65 years with mild to moderate renal insufficiency	CIN, ΔScr≥0.5mg/dL or≥25% within 48h post-catherization	case	109	17
									control	121	7
Dangas G	2005	Caucasian	Israel	retrospective	ACEI	observational	CKD and non-CKD	CIN, ΔScr≥0.5mg/dL and/or≥25% at 48h post-catherization	case	1957	313
									control	5273	756
Gupta RK	1999	Indian	India	prospective	Captopril	RCT	DM with Scr≤6mg/dL	CIN, ΔScr>0.5mg/dL at 24h post-catherization	case	35	2
									control	36	10
Hashemi M	2005	Caucasian	Iran	prospective	Captopril	RCT	Scr≤2mg/dL	CIN, ΔScr>0.5mg/dL at 48h post-catherization	case	42	5
									control	46	5
Kiski D	2010	Caucasian	German	prospective	ACEI/ARB	observational	Scr≥1.3 mg/dL and ≤3.5 mg/dL	CIN, ΔScr≥0.5mg/dL or≥25% within 72h post-catherization	case	269	32
									control	143	6
Li XM	2011	East Asian	China	prospective	Benazepril	RCT	mild to moderate impairment of renal function	CI-AKI, ΔScr≥0.5mg/dL or≥25% within 72h post-catherization	case	52	2
									control	62	6
Oguzhan N	2013	Caucasian	Turkey	prospective	Valsartan	RCT	Scr<2.1 mg/dL	CIN, ΔScr>0.5mg/dL or >25% within 48-72h post-catherization	case	45	8
									control	45	3
Rim MY	2012	East Asian	Korea	retrospective	ACEI/ARB	observational	–	CI-AKI, ΔScr≥0.3mg/dL or≥50% within 48h post-catherization	case	3392	448
									control	1907	116
Rosenstock JL	2008	Caucasian	US	prospective	ACEI/ARB	RCT	CKD stages 3–4	CIN, ΔScr>0.5mg/dL or >25% at 72h post-catherization	case	113	7
									control	63	4
Shemirani H	2012	Caucasian	Iran	prospective	Captopril	RCT	Scr<1.5mg/dL and eGFR >60mL/min	CIN, ΔScr>0.5mg/dL or >25% within 48h post-catherization	case	60	2
									control	60	3
Toprak Ö	2003	Caucasian	Turkey	prospective	ACEI	RCT	Scr<2mg/dL	CIN, ΔScr>0.5mg/dL or >50% at 48h post-catherization	case	48	5
									control	32	1
Umruddin Z	2012	Caucasian	US	retrospective	ACEI/ARB	observational	non-ESRD	CIN, ΔScr>25% within 48h post-catherization	case	92	56
									control	109	40
Wi J	2011	East Asian	Korea	retrospective	ACEI/ARB	observational	–	CI-AKI, ΔScr>0.5mg/dL or>25% within 48h post-catherization	case	874	118
									control	167	30

ACEI: angiotensin-converting enzyme inhibitor; ARB: angiotensin receptor blocker; RCT: randomized controlled trial; eGFR: estimated glomerular filtration rate; CKD: chronic kidney disease; DM: diabetes mellitus; Scr: serum creatinine; ESRD: end-stage renal disease; CIN: contrast-induced nephropathy; CI-AKI: contrast-induced acute kidney injury; ΔScr: an increase in serum creatinine from a baseline value; a: not mentioned;

**Table 2:** The population charcteristics of all qualified studies.

First Author	Status	Age,year	Gender,M(%)	eGFR, ml/min/1.73m^2^	Creatinine, mg/dL	LVEF, %	SBP, mmHg	DBP, mmHg	Contrast volume, ml
Barış N	case	64.1±12.0(ACEI)	28.5	89.3±37.1(ACEI)	0.97±0.33(ACEI)	48.5±12.5(ACEI)	127.7±14.6(ACEI)	79.2±10.5(ACEI)	111.7±38.6(ACEI)
		65.4±13.1(ARB)		83.5±33.3(ARB)	0.97±0.26(ARB)	48.4±12.3(ARB)	128±17.2(ARB)	78.9±12.6(ARB)	111.1±37.1(ARB)
	control	61.9±12.9	28.4	87.9±34.6	1.00±0.32	46.9±13.7	124.4±11.0	76.4±9.5	110.0±25.8
Cirit M	case	71.4±3.9	68.8	50.5±7.8	1.34±0.20	54.69±9.49	128.62±17.88	78.62±13.10	107.12±5.74
	control	71.3±4.0	58.7	50.6±8.9	1.33±0.18	55.52±8.44	125.39±17.03	75.74±10.06	107.03±5.89
Dangas G	case	–^a^	–	–	–	–	–	–	–
	control	–	–	–	–	–	–	–	–
Gupta RK	case	55.8±6.9	91.4	–	1.38±0.27	54.2	139±18	81±11	116.6±11.4
	control	56.0±6.2	88.9	–	1.33±0.18	55.4	137±19	76±12	118.4±9.3
Hashemi M	case	55.1±17	71.4	–	0.98±0.43	–	–	–	225±120
	control	53.6±21	71.7	–	1.05±0.39	–	–	–	223.3±130
Kiski D	case	67.8±10.0	82.5	–	1.61±0.38	–	–	–	–
	control	65.9±10.5	85.3	–	1.55±0.38	–	–	–	–
Li XM	case	60.8±9.2	57.7	70.73±14.20	0.94±0.18	55.6±8.9	129.5±14.4	75.6±12.9	161.37±51.23
	control	61.8±9.4	56.5	70.64±16.38	0.94±0.19	57.2±9.6	131.5±12.6	74.4±12.5	159.90±51.58
Oguzhan N	case	66.4±12.2	60.0	69.19±24.91	1.13±0.33	46.16±14.27	–	–	60(30.0–200)
	control	62.1±8.8	66.7	72.01±19.68	1.07±0.23	49.76±14.99	–	–	60(25.0–250)
Rim MY	case	62.1±12.7	58.0	73.68±31.14	–	56.73±14.48	121.61±22.28	74.18±13.10	157.18±89.35
	control	60.7±12.8	54.0	79.87±24.50	–	58.48±13.59	119.76±17.07	73.72±10.71	146.16±82.01
Rosenstock JR	case	71.8±10.2	54.0	44.6±10.4	1.5±0.4	–	–	–	142±76
	control	68.5±11.9	63.5	44.3±10.6	1.6±0.4	–	–	–	125±75
Shemirani H	case	64.0±11.0	43.3	–	–	–	138±20	72±12	115±57
	control	63.0±15.0	48.3	–	–	–	136±20	76±11	120±40
Toprak Ö	case	58.6±9.9	52.1	–	0.91±0.28	–	130.1±16.65	76.93±12.95	–
	control	57.7±12.0	56.3	–	1.05±0.36	–	131.3±16.21	81.72±14.29	–
Umruddin Z	case	–	–	–	–	–	–	–	–
	control	–	–	–	–	–	–	–	–
Wi J	case	–	–	–	–	–	–	–	–
	control	–	–	–	–	–	–	–	–

eGFR: estimated glomerular filtration rate; LVEF: left ventricle ejection fraction; SBP: systolic blood pressure; DBP: diastolic blood pressure; ACEI: angiotensin-converting enzyme inhibitor; ARB: angiotensin receptor blocker;

^a^:data not available; continuous variables are expressed as mean±SD (standard deviation) or median (5th and 95th percentiles)

### Effects of RAAS blockers treatment on clinical primary outcome

**Fig 2:**
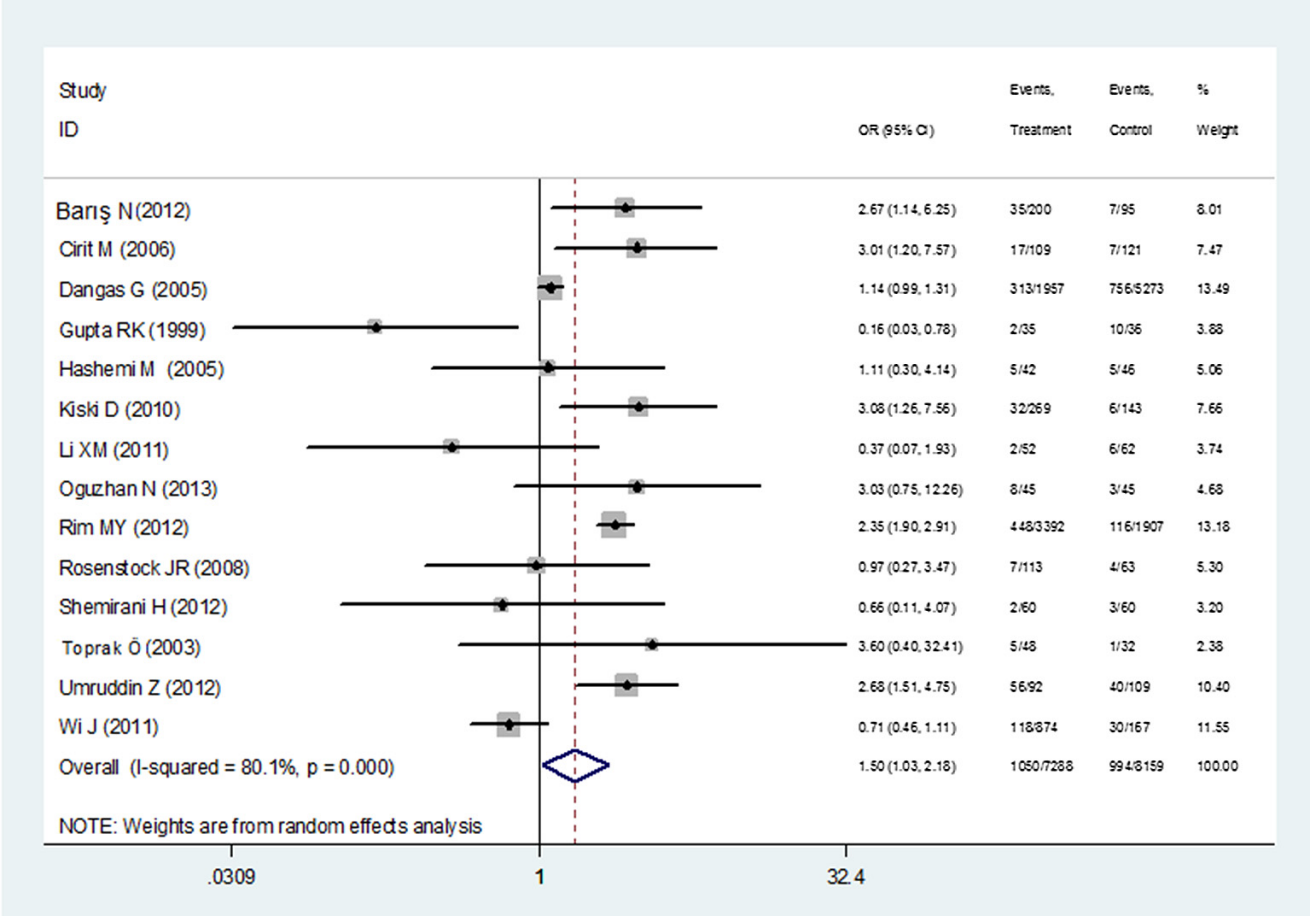
Forest plot illustrating the overall comparison for the CIN incidence among patients receiving ACEI/ARBs versus controls.

**Fig 3:**
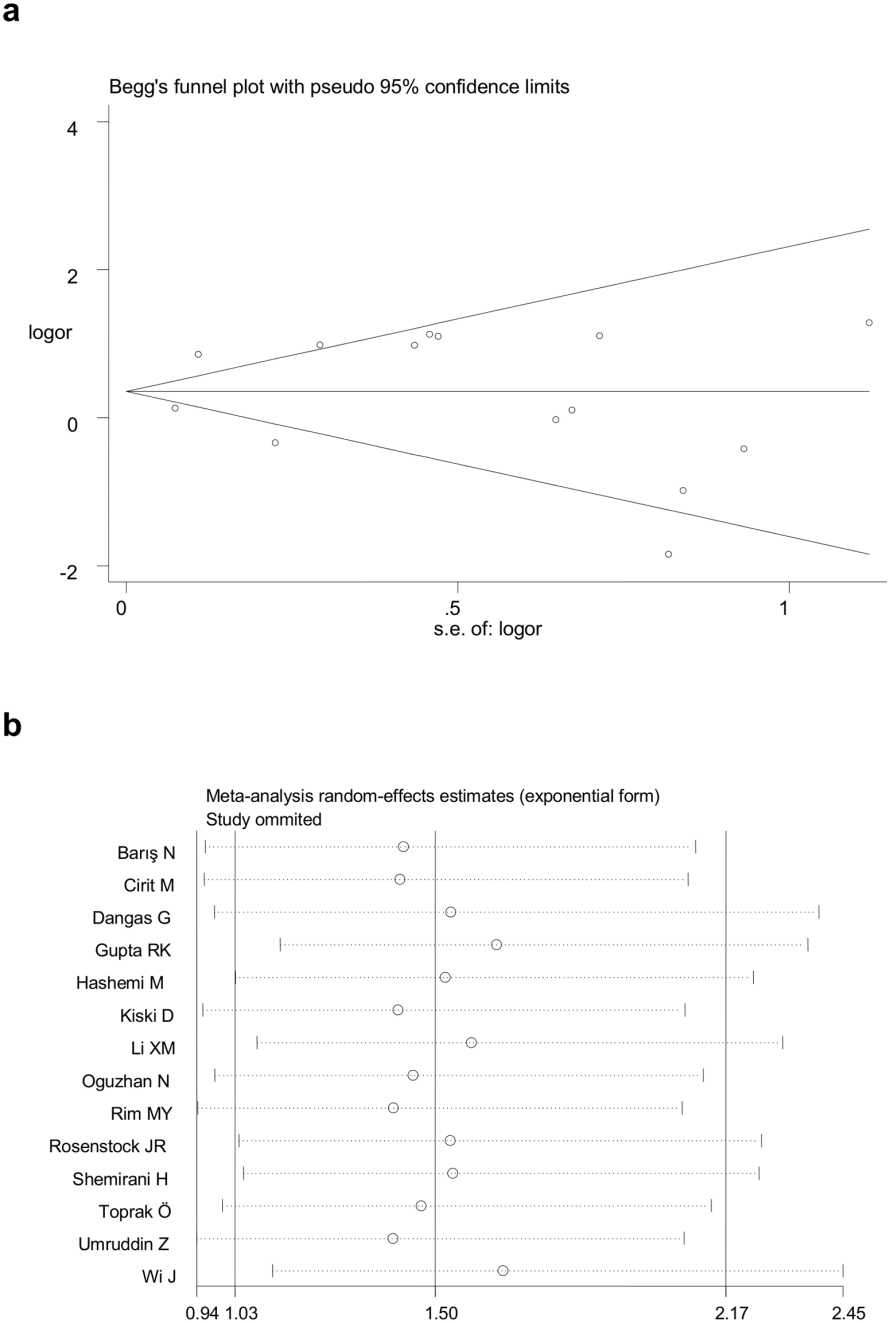
Funnel plot analysis to estimate publication (Fig 3a) and sensitivity analysis for the overall estimate on the association between ACEI/ARBs and CIN (Fig 3b).

The CIN incidence in the ACEI/ARBs group was 14.4% (n = 1050) versus 12.2% (n = 994) in the control group. The overall estimate based on the random-effects model demonstrated that the administration of ACEI/ARBs was significant with the increased risk of CIN in patients undergoing CAG, with the ACEI/ARBs group showing a 50% risk increase of CIN (95%CI: 1.03–2.18, P = 0.03, Fig 2). There was strong evidence of between-study heterogeneity in the overall comparison (I^2^ = 80.1%, P_heterogeneity_ <0.001). There was no evidence for publication bias as reflected by the relatively symmetric funnel plots and the Egger’s test (t = 0.20, P = 0.85). A sensitivity analysis was performed to detect which study, if any, had the source of the greatest between-study heterogeneity. No individual study was identified as the cause for the heterogeneity in the total analysis (Fig 3).

### Quality Assessment

Assessment of risk of bias of all the eligible studies was summarized in Tables 3 and 4. Of all the 7 RCTs, 2 studies were judged to be at low risk of bias [36, 37], 2 at high risk of bias [15, 39] and 3 had an unclear risk of bias [38, 40, 41]. As for the 7 observational studies, 6 studies (86%) scored 5 stars or more, suggesting a moderate to good quality.

**Table 3:** Risk of bias assessment of randomized controlled trials using the Cochrane Collaboration’s tool.

First Author		Selection bias		Performance bias	Detection bias	Attrition bias	Reporting bias	
	Year	Random sequence generation	Allocation concealment	Blinding of participants and personnel	Blinding of outcome assessment	Incomplete outcome data	Selective reporting	Other bias
Gupta RK	1999	Low	Low	Low	Low	Low	Low	Low
Hashemi M	2005	Low	Low	Low	Low	Low	Low	Low
Li XM	2011	Low	Low	High	Unclear	Low	High	Low
Oguzhan N	2013	Low	Low	Unclear	Unclear	Low	Low	Low
Rosenstock JL	2008	Low	Unclear	High	Unclear	Low	Low	Low
Shemirani H	2012	Low	Unclear	Unclear	Unclear	Low	Low	Low
Toprak Ö	2003	Low	Unclear	Unclear	Unclear	Low	Low	Low

Low: low risk of bias; High: high risk of bias; Unclear: unclear risk of bias

**Table 4:** Risk of bias assessment of non-randomized controlled trials according to the Newcastle-Ottawa Scale.

			Quality Indicators	
First Author	Year	Selection	Comparability	Exposure
Barış N	2012	☆☆☆	☆☆	☆☆
Cirit M	2006	☆☆☆	☆☆	☆☆☆
Dangas G	2005	☆☆	☆	☆
Kiski D	2010	☆☆☆	☆☆	☆☆
Rim MY	2012	☆☆☆	☆☆	☆
Umruddin Z	2012	☆☆	☆☆	☆
Wi J	2011	☆☆☆	☆☆	☆

### Cumulative analysis and meta-regression analysis

There was no significant evidence suggesting that the results of the first published study triggered ensuing replication found in the cumulative meta-analysis (data not shown). To identify potential factors that may influence the difference in effect size between the ACEI/ARBs and the control groups, we conducted a univariate meta-regression analysis by incorporating various trial-level covariates including ethnicity, study design, study type, male percent, the average levels of age, baseline renal function, baseline blood pressure and CM volume. There was no evidence indicating these above factors contributed to the between-study heterogeneity.

### Evidence from randomized controlled trials

**Fig 4:**
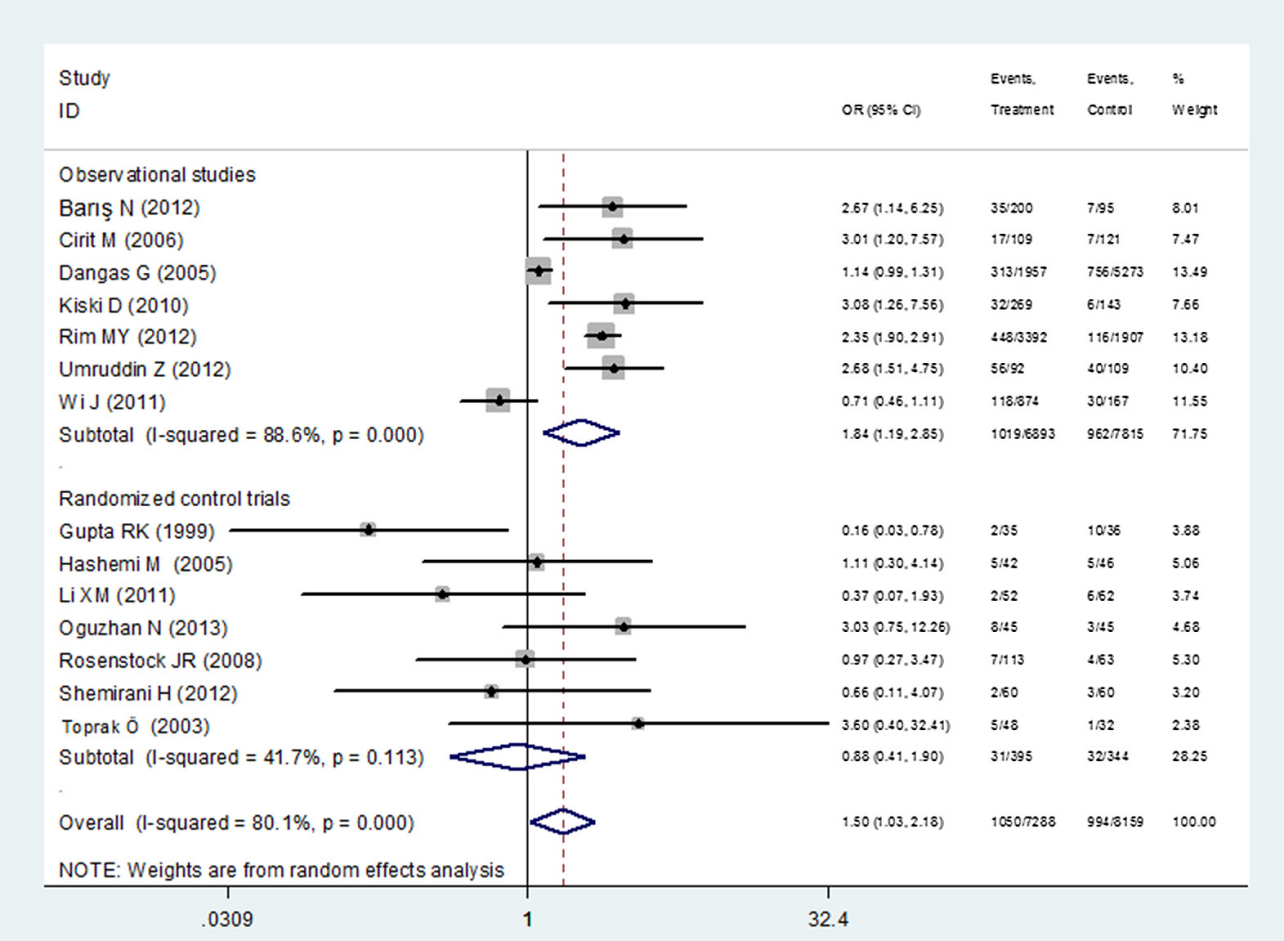
Forest plot for the association between ACEI/ARBs and CIN when data were stratified into the RCTs and the observational studies.

In the 7 RCTs, the CIN incidence of ACEI/ARBs group (7.85%, n = 31) was similar to that among controls (12.2%, n = 32). The administration of ACEI/ARBs was not associated with CIN risk (P = 0.74, OR = 0.88, 95%CI 0.41–1.90) without between-study heterogeneity (I^2^ = 41.7%, P_heterogeneity_ = 0.11) (Fig 4). There was no evidence for publication bias according to the Egger’s test (t = -0.13, P = 0.90). The meta-regression analysis suggested that ethnicity contributed to a source of heterogeneity (P = 0.048). Subgroup analysis found that the administration of ACEI/ARBs was not associated with CIN among Caucasians (P = 0.33, OR = 1.40, 95%CI 0.72–2.74, I^2^ = 0%, P_heterogeneity_ = 0.56). Using a random-effects model, the pooled mean differences in eGFR (P = 0.87, SMD = -0.031, 95%CI -0.41–0.35, P_heterogeneity_ = 0.039, I^2^ = 69.1%), in Scr (P = 0.81, SMD = -0.049, 95%CI -0.45–0.35, P_heterogeneity_ = 0.001, I^2^ = 79.8%) and in BUN (P = 0.50, SMD = -0.115, 95%CI -0.45–0.22, P_heterogeneity_ = 0.157, I^2^ = 45.9%) between the ACEI/ARBs and the control group were not significant.

### Evidence from the observational studies

Seven observational studies including 14,708 patients (6,893 in the ACEI/ARBs group and 7,815 in the control group) were analyzed. The CIN incidence in the ACEI/ARBs group was 14.8% (n = 1019) versus 12.3% (n = 962) in the control group. The magnitude of the association in observational studies was remarkably reinforced with the ACEI/ARBs conferring a significant increased risk of CIN (P = 0.006, OR = 1.84, 95%CI 1.19–2.85, P_heterogeneity_ < 0.001, I^2^ = 88.6%) (Fig 4). There was no evidence for publication bias as reflected by the Egger’s test (t = 1.01, P = 0.36). Not any confounding factors described above were responsible for the between-study heterogeneity. Further subgroup analysis by ethnicity suggested that a significantly larger increase in CIN risk due to ACEI/ARBs was found in the Caucasian group (P = 0.007, OR = 2.21, 95%CI 1.24–3.93, P_heterogeneity_ = 0.001, I^2^ = 78.9%) compared to that in the East Asian group (P = 0.647, OR = 1.32, 95%CI 0.41–4.24, P_heterogeneity_ < 0.001, I^2^ = 95.6%).

**Fig 5:**
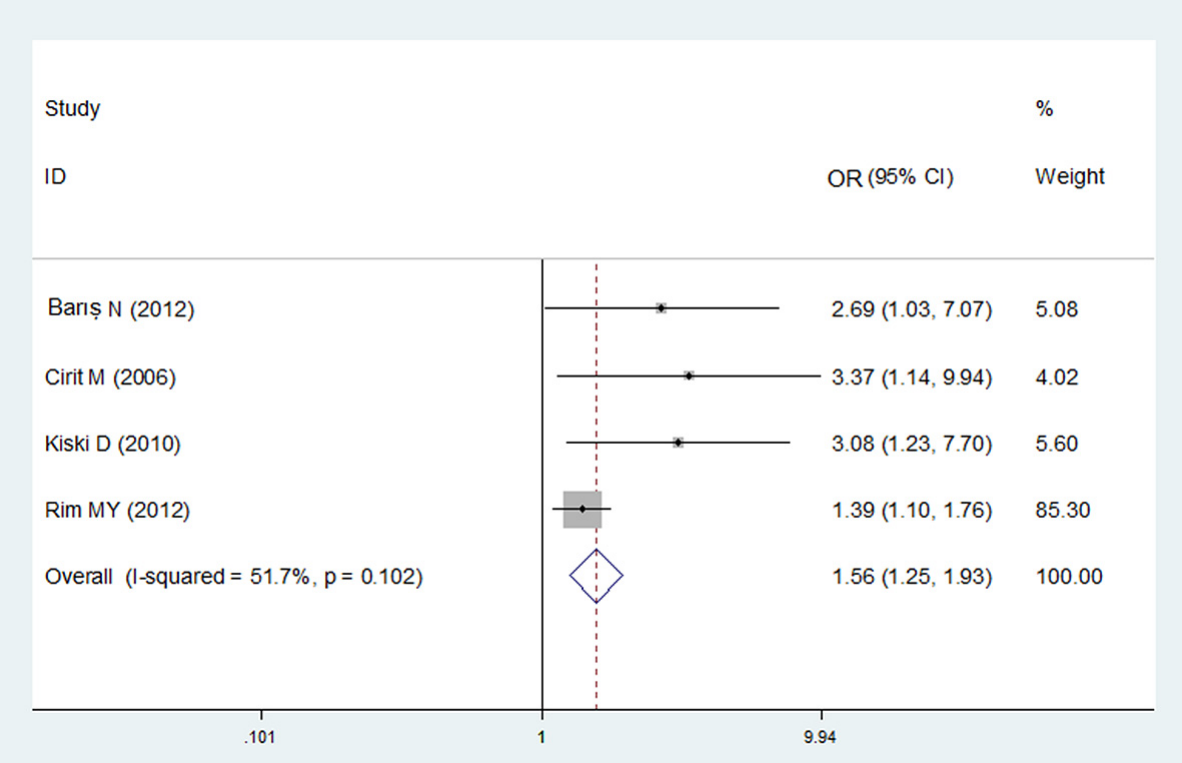
Forest plot for the adjusted OR for the association between ACEI/ARBs and CIN in the observational studies.

To reduce the selection bias, we extracted the adjusted ORs provided in 4 observational studies [16, 18, 19, 35] and pooled the adjusted ORs for further analysis. The adjusted factors differed in the 4 studies but commonly included age, contrast amount, renal function index and diabetes. The combined OR was 1.56 (95%CI 1.25–1.94, P<0.0001), which was weaker than the unadjusted OR (Fig 5).

## Discussion

The proportion of patients undergoing CAG and subsequent CIN is increasing progressively. It is likely to pose new challenges for the clinical cardiologists that refer to the effectiveness and safety of cardiovascular drugs on preventing CIN. There is still lack of robust and consistent evidence that some cardiovascular and reno-protective drugs decrease the incidence of CIN. Paradoxical conclusions exist among clinicians as to whether the administration of ACEI/ARBs should be continued or discontinued prior to CM exposure. Our study provided updated and comprehensive information in relation to the association between the administration of ACEI/ARBs and CIN, as well as the effect of ACEI/ARBs on post-procedural changes of renal function index in patients undergoing CAG, albeit with heterogeneity. Our study is consistent with a harmful effect of ACEI/ARBs on the risk of CIN. However, the positive association was merely observed in the observational studies. We still cannot determine the causal association between ACEI/ARBs and CIN in patients undergoing CAG.

The pathophysiology of CIN remains unclear. Two potential mechanisms contribute to the development of CIN: (1) CM- induced vasoconstriction; (2) direct toxicity to nephrons. Intrarenal vasoconstriction is a vascular response to the Intra-arterial infusion of CM and lead to a reduction in blood flow to the medulla [47]. The medullary ischemia results in a hypoxic-ischemic injury to the kidney with enhanced inflammation and reactive oxygen species. Direct cytotoxicity appears to be related to vacuolization in tubular epithelial cells and cell death [48].

We found the administration of ACEI/ARBs was not associated with change in eGFR, Scr and BUN, most likely due to relatively small sample sizes and insufficient statistical power because few studies measured the post-procedural changes in renal function index both in the ACEI/ARBs group and in the control group. A previous study indicated that the combination of ACEI and furosemide was a risk factor for CIN because it significantly decreased GFR and increased proteinuria in patients with diabetes and hypertension [49]. Further data from large-scale and well-designed studies are needed to precisely estimate the effect of ACEI/ARBs on the changes of renal function.

A previous meta-analysis reported ACEIs were not associated with CIN [50]. Actually, this study by Li [50] included a Chinese population undergoing intravenous urogram, rather than CAG. In addition, there was no evidence indicating that an abstract [51] and an individual study [41] by the same authors involved in their meta-analysis were independent of each other or derived from two different populations [41, 51]. The effect size was probably misestimated because of overlapped data. In addition, the sample size of their study is relatively small (792 participants undergoing intravascular angiography).

Our study included large scale studies published recently (7,288 patients received ACEI/ARBs while 8,159 patients received placebo or were naive to ACEI/ARBs), serving to potentially challenge some of the concerns that the administration of RAAS blockers protects against the development of CIN. However, the effect of RAAS blockers appears heterogeneous and relatively modest with uncertain clinical effects.

It is too early to judge whether ACEI/ARBs should be withdrawn prior to catheterization. Given RAAS blockers play an integral role in the treatment of cardiovascular diseases and have a significant benefit in patients with congestive cardiac failure, hypertension and myocardial ischemia, it is wise to carefully monitor the administration of ACEI/ARBs in patients at high risk for CIN. Likewise, it is also not recommended to start ACEI/ARBs before CAG or PCI solely for the reason of reducing the CIN risk based on current evidence.

Actually, the effective strategy to prevent CIN remains unclear. Some drugs including N-acetylcysteine (NAC), theophylline, sodiumbicarbonate and statins were reported to be potentially protective to CIN [52]. However, the efficacy of these drugs appears heterogeneous and modest [53]. Statins possess high anti-inflammation and anti-oxidant effects on renal injury and their protective effect were dose-dependent. Atorvastatin and rosuvastatin at high dose, but not at low dose, significantly decreased the CIN incidence [54]. A network meta-analysis found that non-ionic low-osmolar CM had heterogeneous effects on the risk of CIN. Iodixanol, iomeprol, iopamidol and ioversol had a similar renal safety profile to non-ionic dimer iodixanol while iohexol and ioxaglate had a more harmful effect on renal functions [55].

There are still some limitations in our study. First, our data focus on transitory, surrogate primary outcomes (e.g. CIN incidence and change in renal function) rather than hard clinical outcomes such as acute renal failure requiring dialysis, development of end-stage renal disease, heart failure and in-hospital mortality. In fact, few studies have been designed to investigate the effect of RAAS blockers on these patient- centered outcomes in patients undergoing CAG. Second, there were several different types of ACEIs and ARBs used in the individual studies. Therefore, we cannot identify whether some types of ACEIs and ARBs might have a greater influence on the CIN risk than others. Some studies have demonstrated that differences in GFR decrease exist between patients receiving ACEIs and those who receiving ARBs [56, 57]. ACEIs probably have a different effect on CIN compared to ARBs and further studies with uniform protocols are required to address this issue. Third, we focused on the administration of ACEI/ARBs. We could not rule out the impact of other moderators of CIN such as the usage of statins and the type of CM because few studies reported this information. Therefore, a large, well-designed study is needed to investigate the interaction of ACEI/ARBs with other risk factors of CIN. Fourth, the sources of ethnical and methodological between-study heterogeneity were identified in our study. We need to interpret the results cautiously. Although there was no evidence of publication bias, we could out completely exclude the influence of confounding factors. The summary adjusted and unadjusted ORs in the observational studies suggested a potential adverse effect of ACEI/ARBs on CIN but not in the RCTs. This can be explained by the fact that the sample size of the RCTs (739 patients) was much smaller than the observational studies (14,708 patients). A lack of high-quality RCTs resulted in insufficient statistical power. Another explanation was that the added weight was given to small observational studies with large effects, resulting in an overestimated risk of CIN in patients using ACEI/ARBs. Theoretically, observational studies are more susceptible to bias compared to the RCTs because the assignment to the exposure in observational studies is not randomly allocated. The potential confounding is unequally distributed in each group and may be considered as a possible threat to validity. Larger RCTs are urgently needed to explore the effect of ACEI/ARBs on the CIN incidence.

In conclusion, we merely found a potential adverse effect of continuation of RAAS blockade on CIN at the time of coronary angiography in the observational studies. Much caution must be used in interpreting this finding because the robustness of our study remains weak. Large clinical trials incorporating the evaluation of clinically patient-centered outcomes with different potential risks of CIN are needed to confirm the robustness of our results and to better determine the clinical utility of pharmaceutical strategies for the CIN prevention.

## Supporting Information

S1 Table PRISMA checklist for this meta-analysis. (DOC)
